# Luteal Phase Support in IVF: Comparison Between Evidence-Based Medicine and Real-Life Practices

**DOI:** 10.3389/fendo.2020.00500

**Published:** 2020-08-18

**Authors:** Federica Di Guardo, Habib Midassi, Annalisa Racca, Herman Tournaye, Michel De Vos, Christophe Blockeel

**Affiliations:** ^1^Centre for Reproductive Medicine, Universitair Ziekenhuis Brussel, Vrije Universiteit Brussel, Brussels, Belgium; ^2^Department of General Surgery and Medical Surgical Specialties, Gynecology and Obstetrics Section, University of Catania, Catania, Italy; ^3^Polyclinique Ibn Annafis, Faculte de Medecine de Sfax, Universite de Sfax, Sfax, Tunisia

**Keywords:** luteal phase support, progesterone, IVF, ICSI, ART, reproductive endocrinology, pregnancy, real-life practices

## Abstract

**Background:** Luteal phase support (LPS) in assisted reproduction cycles has been widely investigated in recent years. Although progesterone represents the preferential product for luteal phase supplementation in cycles with fresh embryo transfer, there is ongoing debate as to when to start, which is the best route, dosage and duration, and whether there is a place for additional agents. Nevertheless, fertility specialists do not always adhere to evidence-based recommendations in their clinical practice. The aim of this worldwide web-based survey is to document the currently used protocols for luteal phase support and appraisal tendencies of drug prescription behavior and to compare these to the existing evidence-based literature.

**Material and Methods:** A questionnaire was developed and sent by secure e-mail to 1,480 clinicians involved in ART worldwide. One hundred and forty-eighth clinicians from 34 countries returned completed questionnaires.

**Results:** Progesterone support is usually started on the day of oocyte retrieval. Eighty percent of clinicians applied the administration of vaginal progesterone only. Intramuscular progesterone was prescribed by 6%, while oral progestin or subcutaneous progesterone were each prescribed by 5% of clinicians, respectively. Progesterone was administered until 8-10 weeks' gestation by 35% and 12 weeks by 52% of respondents.

**Conclusions:** Vaginal administration was the preferred route for luteal phase support. The reported emerging use of the oral route confirms the expected shift in clinical practice as a result of recent evidence showing a reassuring safety score of oral progestins. In spite of the lack of evidence supporting the continuation of luteal support until 12 weeks' gestation, this practice was adhered to by more than half of the clinicians surveyed, highlighting the difference between evidence-based medicine and real-life practices.

## Introduction

The luteal phase is defined as the period between the ovulation day and the onset of menses 2 weeks later, or the establishment of a pregnancy (1). This phase plays a crucial role in the development of a pregnancy preparing the endometrium for the blastocyst implantation. When pregnancy occurs, human chorionic gonadotrophin (hCG) is first produced by the blastocyst maintaining the corpus luteum and its secretions (2), until the start of placental steroidogenesis during the fifth gestational week (3), an event referred to as the luteoplacental shift. The importance of hormonal supplementation during the luteal phase has been recognized from the early years of reproductive medicine (4): strong scientific evidence suggested that the luteal phase is deficient in almost all patients who undergo ovarian stimulation for IVF (5–7). This luteal phase defect results in altered endometrium development causing desynchrony between the phase of the endometrium and the cleaving embryo (6, 8–11). The past 10 years have seen a wealth of studies investigating the efficiency, route and duration of luteal phase support in fresh ART cycles. According to a recent Cochrane meta-analysis, progesterone administration resulted in higher live birth rates compared to placebo or no treatment (12), whereas hCG administration for luteal phase support (LPS) appeared not to have any beneficial effect on live birth rate compared to progesterone and compared to a combination of progesterone and estrogens; instead LPS with hCG was associated with a higher OHSS rate (12–14).

Several studies have focused on the route and hormonal dosage of different LPS formulations (15–17). As suggested by the European Society of Human Reproduction and Embryology (ESHRE) guidelines, the daily administration of 50 mg of intramuscular progesterone, 25 mg of subcutaneous progesterone and 600 mg of micronized vaginal progesterone may be equally efficient (18). With regard to the timing of LPS, progesterone supplementation should be started in the interval between the evening of the day of oocyte retrieval and day 3 post oocyte retrieval (19–23) while starting progesterone before oocyte retrieval should be discouraged (24).

Finally, according to a meta-analysis including 6 RCTs investigating the duration of progesterone administration, no significant difference in live birth rate was found between patients who discontinued progesterone at the time of the pregnancy test and those who continued progesterone administration until week 6/7, which indicates that generalized progesterone supplementation beyond the first positive pregnancy test may not be necessary (25). In view of this, ESHRE developed a recommendation for clinical practice suggesting that progesterone administration for LPS should be continued at least until the day of the pregnancy test (18).

Nevertheless, there is great variation in the duration of LPS in clinical practice: some clinicians discontinue LPS on the day of a positive hCG test, whereas others prefer to continue LPS until 12 weeks of pregnancy. Interestingly, there appears to be the tendency toward prolonging progesterone supplementation until 8–10 weeks following conception, in spite of a lack of available evidence in support of such a practice (26).

In view of this, we set out to conduct a survey aiming to document the implementation of LPS protocols in clinical practice and to appraise tendencies of drug prescription behavior.

## Materials and Methods

We conducted a two-phase web-based survey sent to gynecologists/reproductive endocrinologists involved in ART. In the first phase of the survey (April-May 2017), the questionnaire was sent to clinicians based in Tunisia. In the second phase (August-October 2018), we decided to expand the survey internationally and to include clinicians involved in ART outside Tunisia, in order to increase the generalisability of the study data.

All clinicians surveyed were members of ESHRE, world-renowned *in vitro* fertilization (IVF) practicing gynecologists and authors of papers in Q1-2 scientific reproductive medicine journals. The survey consisted of four questions regarding the use of progesterone for luteal phase supplementation in IVF/ICSI cycles. Questions are listed in Table 1.

**Table 1:** Questions.

1. When do you start the luteal-phase support in IVF/ICSI cycles?
2. Which agent/route do you use?
3. Which progesterone dosage do you use?
4. How long do you continue the administration of progesterone during early pregnancy?

*Questions were sent directly to the responsible of the IVF centers or to the clinicians via secure mail*.

The questionnaires were sent via secure email and responses were collected securely by email or telephone contact. Invited participants who did not respond to the survey were not contacted again and no incentives were provided for participation. Data from both study phases were included for analysis.

### General Data Protection Regulation (GDPR)

Our survey was conducted in agreement with the GDPR privacy policy. The survey only contains names and e-mail addresses of clinicians, patients' personal identifying information was excluded. The clinicians' personal identifying information and the survey data were securely stored in a dedicated database protected by password. These data will not be shared with any third parties.

### Survey Sample Size Calculation

According to Bartlett et al. (27), in order to obtain a sample of 110 subjects, considering a margin of error of 0.03 and alfa level of 0.05, a sample size of 1,500 was required. By interviewing 1,480 clinicians, we obtained 148 answers. Given this, we considered that our sample was appropriate to drag robust conclusions.

## Results

Between April-May 2017 and August-October 2018, 1,480 clinicians received the survey. Of those, 148 from 34 different countries returned completed questionnaires yielding a response rate of 10%. The respondents were distributed as follows: 24 were based in Tunisia and there were 124 clinicians from 33 other countries worldwide. The geographical distribution of respondents is displayed in Supplementary Figure 1.

## Questions

1. When do you start luteal-phase support in IVF/ICSI cycle for fresh embryo transfer following hCG triggering?

Seventy one percent (105/148) of the clinicians prescribed progesterone administration to their patients from the day of the oocyte retrieval onwards. LPS using progesterone was initiated on the day after the oocyte collection by 23.6% (35/148) of doctors, while 3.38% (5/148), and 2.02% (3/148) started the progesterone supplementation 2 and 3 days after egg collection, respectively.

2. Which agent/route do you routinely use?

Eighty percent (119/148) of the clinicians preferred to use the vaginal progesterone alone as LPS; 4% (6/148) of them used vaginal progesterone in association with intramuscular or oral progesterone. Intramuscular progesterone alone, was used by 6% (9/148) of doctors and oral progesterone by 5% (7/148) of doctors. The usage of the subcutaneous progesterone was reported by 5% (7/148) of clinicians (Table 2).

**Table 2:** Distribution of progesterone routes according to the number of clinicians and dosage.

**Progesterone routes**	**Clinicians%(*n*)**	**Dosage (mg/day)**
Vaginal	80% (119/148)	600 mg/d (103/119) 200/400 mg/d (16/119)
Vaginal + OS	2% (3/148)	600/400 mg/d +20–30 mg/d (3/148)
Vaginal + IM	2% (3/148)	600/400 mg/d + 50 mg/d (3/148)
IM	6% (9/148)	50–100 mg/d (9/148)
Subcutaneous	5% (7/148)	25 mg/d (7/148)
OS	5% (7/148)	20–30 mg/d (7/148)

*OS: oral, IM: intramuscular. Vaginal route agent: micronized progesterone tablets 200 mg*.

3. Which progesterone dosage do you use?

Considering the total number of clinicians recurring to vaginal progesterone as LPS (80%; 119/148), the most used formulation and dose for vaginal route were the vaginal tablets at doses of 600 mg/d (69.3 %; 103/119).

Lower doses of 200 or 400 mg/d were used by 10.7% (16/119) of doctors.

Intra-muscular progesterone was equally used in dosage of 50/100 mg/d (8%; 12/148). Subcutaneous in dosage of 25 mg/d (5%; 7/148) and oral dydrogesterone in dosage of 20–30 mg/d (7%; 10/148) (Table 2).

4. How long do you continue the administration of progesterone during early pregnancy?

Six percent (9/148) of doctors continued progesterone administration until the pregnancy was confirmed by a positive hCG and discontinued thereafter. Seven percent (10/148) of clinicians discontinued the administration of progesterone after the identification of a fetal heartbeat, whereas 22% continued the LPS until 7–8 weeks (33/148) and 13% (19/148) until 10 weeks. More than half of the clinicians surveyed (52%; 77/148) discontinued LPS only after 12 weeks of gestation (Table 3).

**Table 3:** Comparison between EBM on LPS and Survey results.

**P-LPS**	**EBM**	**Survey results % (*n*)**
Initiation of administration	OR0–OR+3	OR0 71 % (105/148) OR+1 23.6% (35/148) OR+2 3.38% (5/148) OR+3 2.02% (3/148)
Routes and dosages	Vaginal Micronized P IM P 50 mg/d Subcutaneous P 25 mg/d OS Dydrogesterone 30 mg/d	600 mg/d or 200/400 mg/d 80% (119/148) 50–100 mg/d 6% (9/148) 25 mg/d 5% (7/148) 20/30 mg/d 5% (7/148) Combined regimen: Vag + OS/IM 4% (6/148)
Discontinuation of administration	At least until the pregnancy test	PT 6% (9/148) US 7% (10/148) 7/8 weeks 22% (33/148) 10 weeks 13% (19/148) 12 weeks 52% (77/148)

*Number of respondent doctors are expressed as percentage (%) and number (n)*.

*P: Progesterone; LPS: luteal phase support; EBM: evidence-based medicine; OR0: oocytes retrieval evening; OR+1: one day after oocytes retrieval; OR+2: two days after oocytes retrieval; OR+3: three days after oocytes retrieval; IM: intramuscular; OS: oral; Vag: vaginal, mg/d: mg per day, PT: pregnancy test; US: ultrasound with detection of hearth activity. EBM data are based on the latest ESHRE guideline on ovarian stimulation: “Group EREG.OVARIAN STIMULATION FOR IVF/ICSI. Guideline of the European Society of Human Reproduction and Emnbriology.2019”*.

## Discussion

The results of this survey indicate that: (i) most clinicians (71%) start progesterone LPS on the day of egg collection; (ii) the vaginal progesterone administration represents the clinicians' route of first choice (80% of doctors), used alone or in combination with intramuscular progesterone or oral progesterone; (iii) hCG use for LPS has been completely abandoned; (iv) 10% of clinicians prescribe progesterone support using the subcutaneous route (5%) or the oral route (5%) alone; (v) if pregnancy occurs, more than half of clinicians (52%), continue LPS until 12 weeks. Roughly, the geographical distribution of results appears to be similar worldwide, as displayed in Supplementary Tables 1, 2. The clinical practice behaviors evidenced in our survey are mostly comparable with those illustrated in previous studies (26, 28). Moreover, similarly to our data, the abandoning of hCG as LPS, the use of the vaginal route as preferential route, eventually supported by an additional one, and the tendency toward prolonging the duration of progesterone administration until 8–10 weeks following conception, were noticed in precedent surveys (26, 28).

Although our results are on a par with those previously published (12, 26, 28), we observed that the intramuscular route for the administration of progesterone is still used by a significant percentage of gynecologists. Furthermore, the respondents of our survey reported an increasing uptake of oral dydrogesterone. Finally, it appears that LPS is often continued until 12 weeks of gestation worldwide.

With regard to the initiation of LPS, the current survey shows that almost all clinicians start progesterone supplementation on the day of oocyte retrieval (71%) or on the following day (23.6%). These results agree with a recent study highlighting the possibility of starting the progesterone support either on the day of the oocyte retrieval or on the following day, with comparable outcomes in terms of clinical pregnancy rate (CPR), implantation rate (IR) and live birth rate (LBR) (23).

The favorable clinical outcomes after initiation of progesterone supplementation between the evening of the day of oocyte retrieval and day 3 after oocyte retrieval may be related to the inhibitory function of progesterone on uterine contractility in anticipation of the embryo transfer (ET) (29). However, administration of progesterone before oocyte retrieval has a detrimental impact on outcomes (24).

According to results of a Cochrane meta-analysis, hCG is not superior to progesterone for LPS; furthermore, analysis of pooled data pointed toward a higher risk of ovarian hyperstimulation syndrome when hCG was administered in the luteal phase (12–14).

The reported combination of progesterone via the vaginal route and an additional route, a regimen that has not been supported by any scientific evidence, may be linked to the potentially suboptimal adherence of some patients to the vaginal route; it has been hypothesized that clinicians may decide to add a different route on top of the vaginal one, in order to exclude the possibility of suboptimal administration.

When considering the oral route for progestin LPS, dydrogesterone, an oral synthetic progestin, has been reported to be at least as efficacious as vaginal or intramuscular route in supporting the luteal phase (12, 30). However, these data are in contrast with previous evidence showing an insufficient endometrial decidualization when dydrogesterone is used in the donor oocyte model (31). A recent phase III randomized controlled trial comparing the efficacy, safety and tolerability of oral dydrogesterone to micronized vaginal progesterone, showed a comparable safety profile and patients' tolerability (32, 33). Furthermore, oral dydrogesterone administration has been reported to be patient-friendly, avoiding the discomfort and inconvenience related to intravaginal or intramuscular progesterone routes (32, 33).

In agreement with these observations, it is conceivable that oral dydrogesterone may lead to an important shift in the modalities of LPS in women undergoing ART (32, 34). Nevertheless, based on our results, the reported use of the oral route was still limited, both alone (5 % of respondents) or in combination with the vaginal route (2% of respondents) may, at least partly, be explained by a lack of long-term health studies in offspring conceived in cycles with oral dydrogesterone (18) (Table 3).

Considering the results of our survey, the subcutaneous route deserves to be mentioned since it is applied by 5% of clinicians. Indeed, the administration of subcutaneous progesterone is considered a valuable alternative to the vaginal and intramuscular route showing a similar efficacy and safety profile (16, 18, 35).

With respect to the duration of LPS, several studies support the continuation of progesterone supplementation until the ultrasound detection of fetal heart activity (36–40). Furthermore, robust scientific evidence exists to suggest that LPS should be administered at least until the pregnancy test (18, 25).

In accordance with the recommendation and in analogy to the results of a previous survey (26), our study confirms the clinicians' practice to administer LPS until the pregnancy test and illustrates a tendency to prolong the duration of progesterone administration until 8–10 weeks (35% of respondents) or even 12 weeks of gestation (52 % of respondents) (Table 3). Although there is a lack of scientific evidence supporting the use of LPS prolonged regimen, (25, 36–41), an explanation to this trend in a fresh ART cycle may be adopted from the increasingly used artificial endometrial preparation protocol (the so-called HRT cycle) for frozen embryo transfer, where extended luteal phase supplementation could be required because of the lack of endogenous progesterone production in the absence of a corpus luteum. Nevertheless, the exact reason for the prescription of extended LPS is unknown.

An important strength of the current survey is the inclusion of private ART clinicians which gives a more representative insight into clinical practice worldwide. Although the questionnaire was sent directly to the responsible clinician of the IVF centers and to clinicians in private practice, the possibility of including multiple records from a single IVF unit cannot be ruled out. Moreover, the nature of the questionnaire (open questions) may be responsible for the low response rate which may affect the robustness of our results and conclusions. However, although we couldn't reach the number of 1,500 surveyed, basing on Bartlett et al., (27), we reached the required sample size.

As far as we know, this survey indicates a trend toward extended LPS in ART cycles with fresh ET, which underscores an important difference between the available scientific evidence and clinical practice. Nevertheless, although the data of our survey study illustrate global LPS prescription, they are a mere estimation of real clinical practice because the responses provided in the survey have not been verified by objective data.

Our survey illustrates also that vaginal and intramuscular progesterone for LPS is still most commonly prescribed, although other subcutaneous and oral routes for LPS are starting to emerge.

## Data Availability Statement

The datasets generated for this study are available on request to the corresponding author.

## Ethics Statement

The study was approved by the Ethical Committee of the University of Sfax, Tunisia.

## Author Contributions

FD designed the study and wrote the manuscript. HM is responsible for data collection. AR is responsible for the statistical analysis. MD, HT, and CB critically commented and substantially revised the manuscript. All authors participated in drafting the manuscript and approved the final version.

## Conflict of Interest

The authors declare that the research was conducted in the absence of any commercial or financial relationships that could be construed as a potential conflict of interest. The handling Editor declared a past co-authorship with one of the authors HT.
