# Supplementary material for: Luteal Phase Support in IVF: Comparison Between Evidence-Based Medicine and Real-Life Practices
Source: Front Endocrinol (Lausanne). 2020 Aug 18;11:500. doi: 10.3389/fendo.2020.00500 (PMC7461775; doi:10.3389/fendo.2020.00500)

S.Table 1. LPS routes distribution according to number of doctors for each country

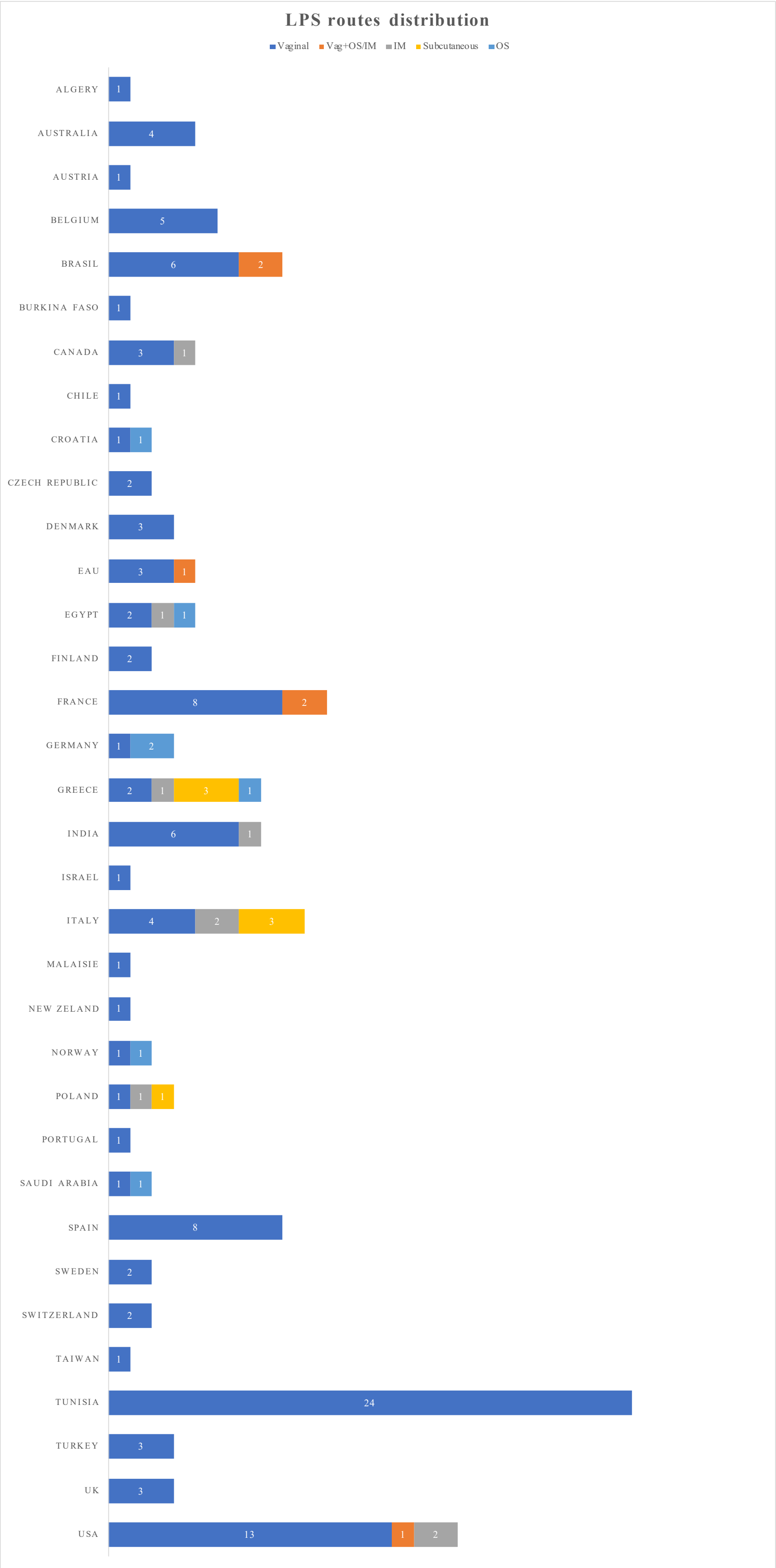

Note. Vag + OS/IM: vaginal + oral or intramuscular, OS: oral, IM: intramuscular

S.Figure 2. LPS discontinuation according to number of doctors for each country

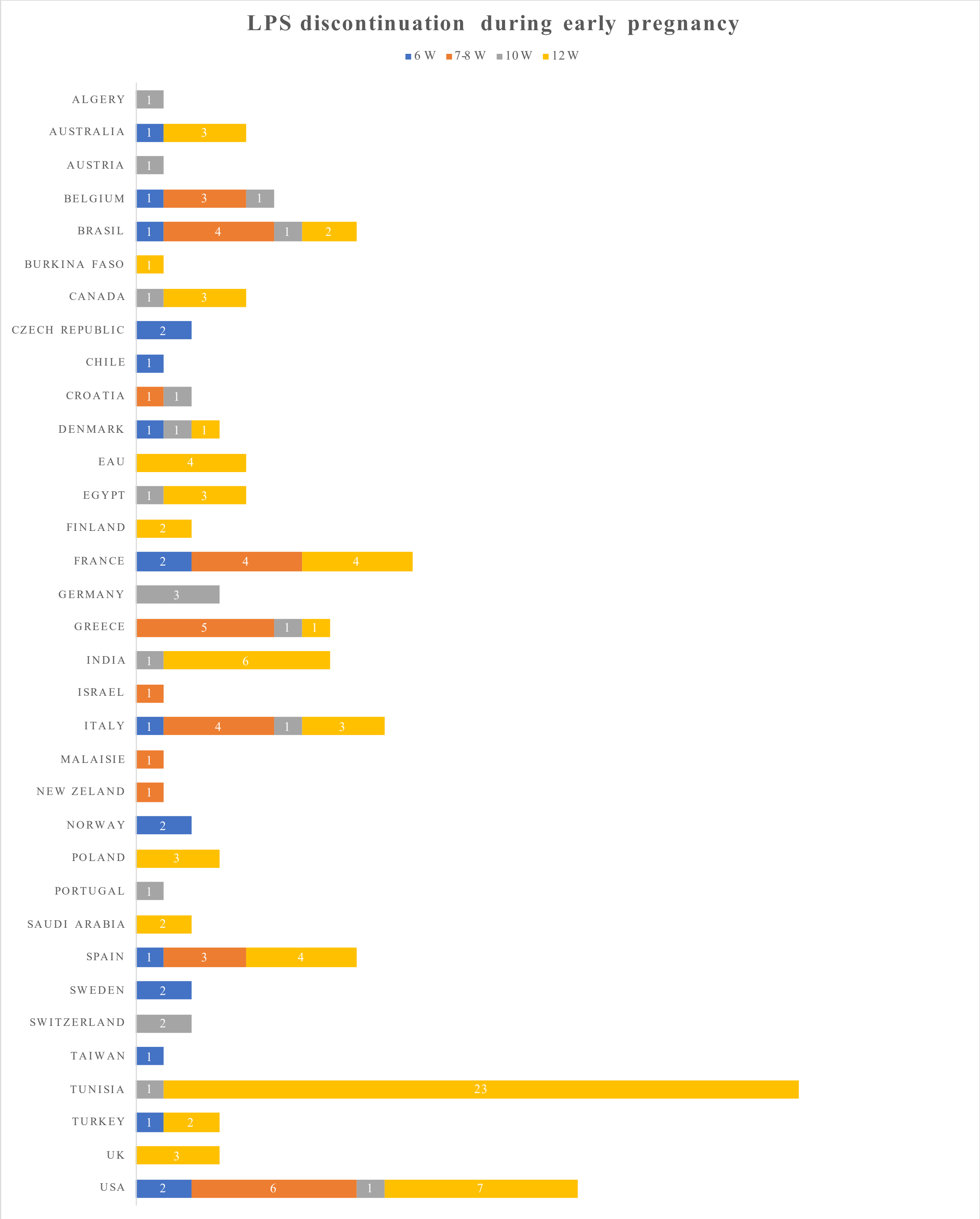

Supplement: Supplementary file 1 [file Data_Sheet_1.PDF]
