# Supplementary figures and images for: Luteal Phase Support in IVF: Comparison Between Evidence-Based Medicine and Real-Life Practices
Source: Front Endocrinol (Lausanne). 2020 Aug 18;11:500. doi: 10.3389/fendo.2020.00500 (PMC7461775; doi:10.3389/fendo.2020.00500)

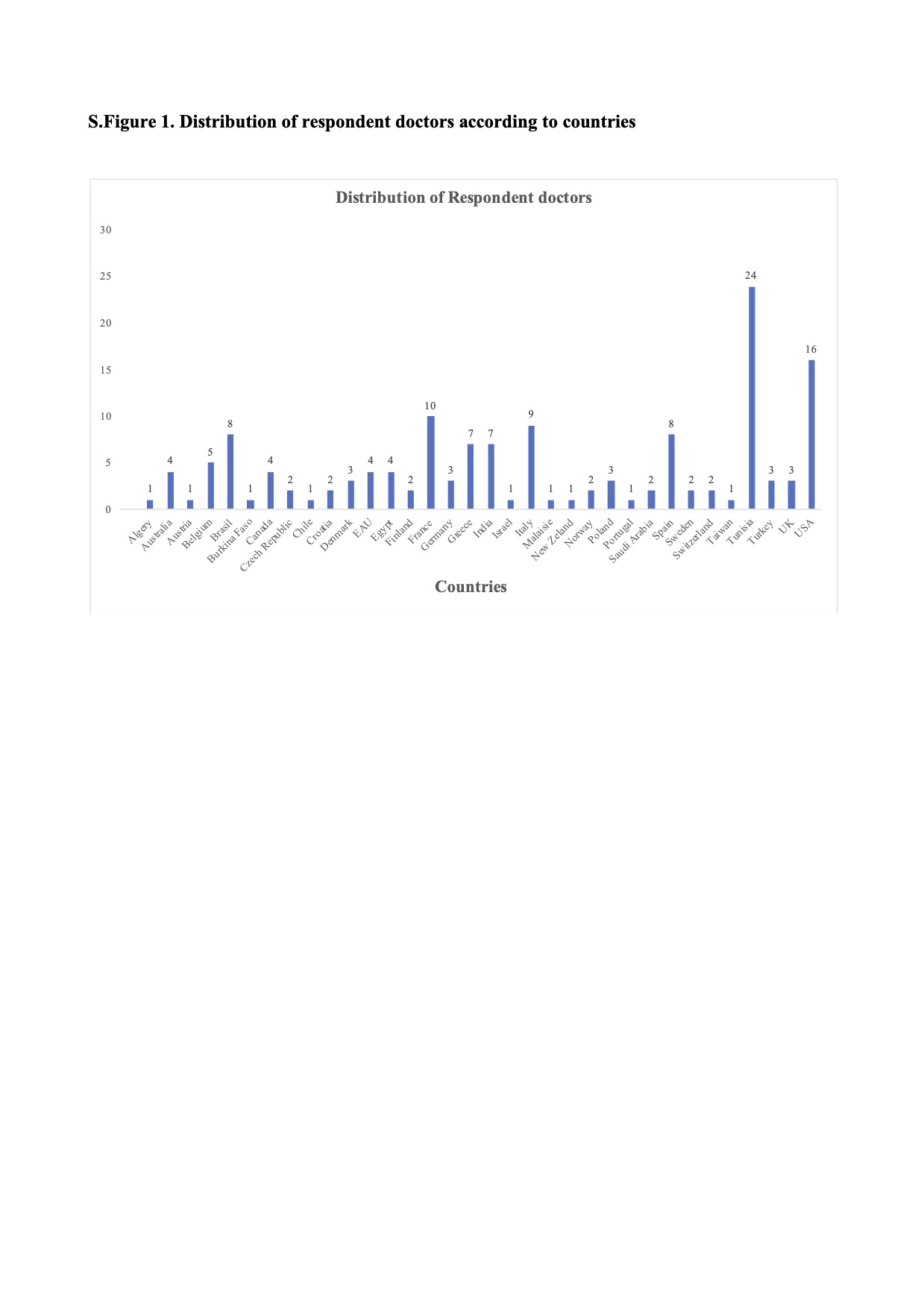

Supplement: Supplementary file 2 [file Image_1.JPEG]
